# Structural Dynamics Analysis of a Large Aperture Space Telescope Based on the Linear State Space Method

**DOI:** 10.3390/s25082476

**Published:** 2025-04-15

**Authors:** Bin Ma, Zongxuan Li, Lin Li, Yunfeng Li, Youhan Peng, Shuhui Ren, Qingya Li, Jiakun Xu

**Affiliations:** 1 Changchun Institute of Optics, Fine Mechanics and Physics, Chinese Academy of Sciences, Changchun 130033, China; binma2020@outlook.com (B.M.); liyunfeng18@mails.ucas.ac.cn (Y.L.); pengyouhan22@mails.ucas.ac.cn (Y.P.); renshuhui@ciomp.ac.cn (S.R.); liqing_ya@163.com (Q.L.); xujiakun@ciomp.ac.cn (J.X.); 2 University of Chinese Academy of Sciences, Beijing 100049, China; 3 Key Laboratory of Space-Based Dynamic Fast Optical Imaging Technology, Chinese Academy of Sciences, Changchun 130033, China; 4 Beijing Institute of Control Engineering, Beijing 100190, China; cast_lilin@163.com

**Keywords:** linear state space, structural dynamics, balance truncation, frequency response analysis

## Abstract

The linear state space model of an optical remote sensing camera with an aperture of φ572 mm was established using the structural dynamics and linear state space theory. Modal reduction was carried out using the balanced reduction method. Combined with the controllable and observability matrix, the model order was reduced. To obtain the frequency response curve between the excitation input point and the response output point, we performed a frequency response analysis with the reduced state space model. The initial frequency response curve was plotted and compared with the response curves of the DC gain method and the balanced reduction method. The accuracy and rationality of the simulation analysis were verified by dynamic tests. The balanced reduction method under state space representation provides a new method for studying the dynamics of lightweight opto-mechanical structures. It can characterize the inherent properties of the system by using the reduction model and has higher computational efficiency, which is helpful to analyze the frequency response characteristics of complex linear systems quickly and accurately.

## 1. Introduction

With the development of space technology, space missions are gradually becoming more and more precise. The requirements for the resolution of camera optical systems and imaging systems are constantly improving. Large aperture, long focal length, high resolution, and lightweight have become the main trends in the development of space optical remote sensing cameras [1,2], which put forward higher requirements for the design and analysis of cameras. In recent years, the ground pixel resolution of cameras has entered the sub-meter level [3]. Hence, the stability of the platform and optical system during on-orbit imaging is increasingly important. As the focal length and aperture of the optical system increase, the stiffness of the optical system will be limited by mass limitation. The camera is becoming more and more sensitive to the micro-vibrations caused by the normal operation of the moving parts on the satellite in orbit. As a result, the wavefront degradation and line-of-sight jitter of the optical system affect the quality of its in-orbit imaging, even causing the entire optical system to fail [4,5,6,7,8,9]. In order to evaluate the dynamic response characteristics and ensure good image quality of the optical system, it is necessary to model and analyze the structural dynamics of the space optical remote sensing camera.

Modal and frequency response analyses are important methods to study the dynamic properties of structures. Modal analysis is the foundation of structural dynamics analysis, which can determine the natural frequency as well as mode shapes so that the structural design can avoid resonance. Frequency response analysis is used to calculate the response of a structure to external excitations. It is the key to structural dynamics analysis. Common methods of system dynamics modeling include the distributed parameter method, discrete coordinate method, mixed coordinate method, modal synthesis method, and finite element method [10]. Among these methods, the finite element method is most commonly used for structural dynamics analysis. Modal analysis and frequency response analysis require the finite element model (FEM). However, for space cameras with complex structures, the system dynamics equations need a high order to ensure the accuracy of the model. To pursue real and accurate analysis results, the FEM is large, and the solution efficiency is low. Model order reduction is closely related to modeling, and it is important to retain the main dynamic features of the original model in the reduced-order model. Therefore, model order reduction has become a critical step in dynamic modeling.

To improve the solution efficiency, scholars have begun to introduce the linear state space method for dynamic modeling [11,12,13]. Jiang et al. applied the modeling techniques in control theory to identify dynamic multi-dimensional models. They conducted modeling experiments to verify the effectiveness of this dynamic modeling method based on the input, output, and transfer conditions [14]. Liu et al. established a state space model of the mirror component through linear state space expression, which used the DC gain method for modal order reduction (MOR). They obtained the frequency response function between the excitation input point and the response output point through the above model [15]. Aleksandar et al. used the balanced reduction method for MOR of the state space model of the spindle, gave the frequency response of the spindle reduction model, and verified the reliability of the reduction model [16]. Ding et al. proposed a new extended state space method for damping systems and calculated the transient dynamic response of structural systems using multiple damping models [17]. Haber et al. proposed a collaborative modeling framework that combines heat transfer principles modeling, experimental verification, finite element analysis, and modal order reduction methods. This framework proved that the thermal dynamics of mirrors can be approximated by a low-order state space model [18].

In modern control theory, state space models can use powerful tools such as vector matrices to deal with problems efficiently with computers. It is an important tool for studying dynamic systems. Therefore, based on the structural dynamics analysis and state space theory, this paper proposed a balanced reduction method for linear state space representation. Dynamic modeling and analysis of an optical camera with an aperture of φ572 mm were carried out. The finite element method was used to analyze the modality of the whole camera, obtain the natural frequency and other modal information of the whole camera, and establish the single-input single-output state space model of the whole camera. The balanced reduction method was used to reduce the system mode for the simplification of the system model. According to the model with balanced reduction, the frequency transfer characteristics were analyzed, and the frequency response curve of the system was plotted. Finally, the accuracy of the simulation analysis was verified by the dynamic test of the whole camera.

## 2. Linear State Space Representation

The state space description of a system is based on states and state spaces. The concepts of state and state variables are more commonly used in the study of control systems, but they have long existed in classical mechanics and some other fields. State space representation can not only reflect the internal state of the system but can also reveal the relationship between the internal state of the system and the input and output. It can use simple models to replace complex models. The output of a dynamic system depends on the system input and intermediate state. The state space approach is well suited to the matrix approach. If the dynamic system is described in a formulaic state space, it is very easy to simulate and find numerical solutions on the computer. The features of state space representations include the following:

(a) Link system outputs to system states and external inputs;
(b) Establish a dynamic time-domain model of the system to realize the internal decoupling of the model;
(c) The short length of the data allows for efficient computation of the problem.

### 2.1. General Form of a State Space Equation

An ordinary differential equation or transfer function model can only describe the relationship between the inputs and outputs of a system. It does not contain all the information about the system, such as internal changes. In order to make up for the shortcomings of the ordinary differential equation or transfer function model and describe the overall motion state of the system, the state space equation and matrix-vector method are used to model the system dynamics.

The dynamic equation for a system with n degrees of freedom (DOF) is as follows:

(1) $$M\ddot{X}+C\dot{X}+KX=Ef\left(t\right)$$

The state variables are chosen to be $x_{1}=x,x_{2}={\dot{x}}_{1}$. Thus, the state variables are

(2) $$x={\left\{\begin{array}{c} x_{1}\\ x_{2} \end{array}\right\}}_{2n\times 1}$$

The *n* DOF dynamic model in physical space requires 2*n* state variables to describe it in state space. According to the given state variables, it can be expressed in the following form:

(3) $$\left[\begin{array}{c} {\dot{x}}_{1}\\ {\dot{x}}_{2} \end{array}\right]=\left[\begin{array}{cc} 0 & I\\ -M^{-1}K & -M^{-1}C \end{array}\right]\left[\begin{array}{c} x_{1}\\ x_{2} \end{array}\right]+\left[\begin{array}{c} 0\\ M^{-1}E \end{array}\right]f\left(t\right)$$

The general form of the state space equation is as follows:

(4) $$\begin{array}{l} \dot{X}=AX+Bu\left(t\right)\\ Y=CX+Du\left(t\right) \end{array}$$

where ***X*** is the state vector, *u* is the input vector, and ***Y*** is the output vector. ***A*** is the system matrix, ***B*** is the input matrix, ***C*** is the output matrix, and ***D*** is the feedback matrix. Combined with the dynamic equations, the following formulation of matrices ***A*** and ***B*** can be obtained:

(5) $$\begin{array}{l} A={\left[\begin{array}{cc} 0 & I\\ -M^{-1}K & -M^{-1}C \end{array}\right]}_{2n\times 2n}\\ B=\left[\begin{array}{c} 0\\ M^{-1}E \end{array}\right],u\left(t\right)=f\left(t\right) \end{array}$$

### 2.2. State Space Model of Optical Remote Sensing Camera

**Figure 1:**
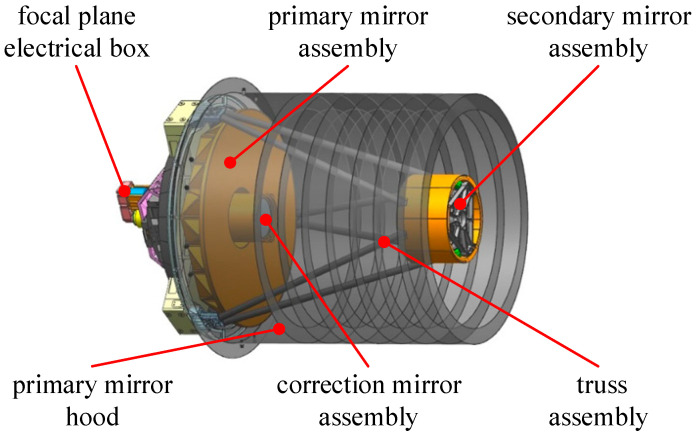
Structural model of optical remote sensing camera with aperture of φ572 mm.

According to the general form of Equation (4), the state space description of the optical remote sensing camera is described. Figure 1 shows an optical camera with an aperture of *φ*572 mm, its focal length is 3500 mm, its resolution is 0.5 m, and the weight of the whole camera is ≤44.5 kg. The structure of the optical camera consists of the primary mirror assembly, secondary mirror assembly, primary mirror hood, correction mirror assembly, truss assembly, focusing mechanism, and focal plane electrical box.

**Figure 2:**
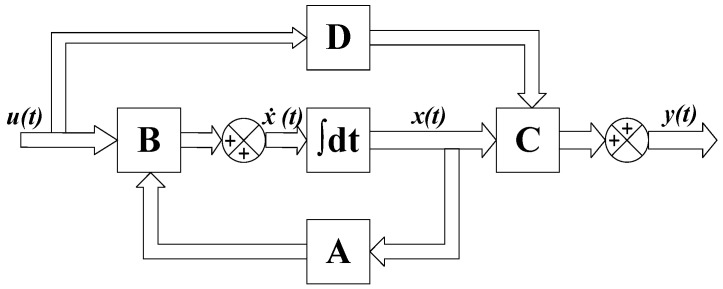
Block diagram of state space system.

The structure of the whole camera is complex. The finite element model has many nodes, but the frequency response analysis only needs the response relationship between the excitation input and output points. Therefore, the optical camera is regarded as a single-input single-output system in the analysis process. Only the modes of the input and measurement nodes are extracted to establish the state space model of the whole camera. The state space model of the whole camera is constructed by extracting only the modes of the input/output nodes.

(6) $$\left\{\begin{array}{l} \dot{x}=Ax+Bu\\ y=Cx+Du \end{array}\right.$$

where the input matrix B represents the coupling relationship between external excitation and the internal state of the camera; and the external input can be various types of excitation signals, such as force, displacement, velocity, and so on. The output matrix C denotes the displacement or acceleration of the key nodes of the camera, which are quantifiable quantities associated with the camera’s state. Matrix C establishes a linear relationship between the system state vector x and the output vector y. The matrix D is not usually used in structural modeling, so the second term of the last expression can be omitted. Figure 2 shows the structure block diagram of the state space model.

## 3. Balanced Reduction Method

The FEM of optical cameras typically contains a large number of degrees of freedom, which generally correspond to specific physical quantities, such as displacements and stresses at structural nodes [19]. In the integrated modeling process, handling such a large model consumes a lot of computational resources and has obvious limitations. Modal reduction can effectively remove unnecessary information from the overall model, thus reducing the model size and improving the efficiency and reliability of integrated modeling.

In order to realize fast matrix computation and data interaction, a linear state space model of an optical camera is established. It focuses on accurately characterizing the dynamic response characteristics of the whole camera. The number of modes is reasonably reduced to obtain a finite number of modes to replace the huge finite element model of the whole camera. To analyze the frequency response, the system model must be concise enough to accurately describe its dynamic performance [20]. To increase the computational speed, the modes should be reduced as much as possible while maintaining the accuracy.

### 3.1. Controllability and Observability

Controllability refers to a state vector that allows for the system to arrive at a specified state from one specified state in a finite time. Observability means that an output vector can be used to predict the initial state of the system in a finite time. Controllability and observability can be precisely described using standard state space notation.

For the above state space model of the whole camera, the controllability is defined as follows:

(1) If the input vector *u* can move the system from some arbitrary state $x_{1}$ to another arbitrary state $x_{2}$ in a finite time, the system is controllable.

(2) Controllability matrix $W_{c}$ can be formed as follows:

(7) $$C=[B\;\;AB\;\;A^{2}B\;\;\ldots \;\;A^{n-1}B]$$

If ***C*** has full rank *n*, the system is controllable. The controllability matrix does not provide the relative controllability of different modes; it shows only whether the whole system is controllable or not. If a mode of the system is not controllable, the system is not controllable.

(3) Another definition of controllability involves the controllability matrix $W_{c}$; the solution of the Lyapunov equation is as follows:

(8) $$AW_{c}+W_{c}A^{T}+BB^{T}=0$$

defined as

(9) $$W_{c}=\int_{0}^{\infty }e^{A\tau }BB^{T}e^{A^{T}\tau }d\tau$$

The system is controllability if the solution $W_{c}(t)$ is non-singular (determinant is non-zero). The diagonal elements of the controllability matrix give information about the relative controllability of the different modes.

The controllability matrix measures the extent to which inputs affect the state of the system [19]. In optical camera analysis, significant values in the controllability matrix indicate that the applied driving force can effectively control the corresponding state variables.

The observability is defined similarly:

(1) If the initial state $x_{0}$ can be obtained by inference from the input vector *u* and the output vector in a finite time (0, *t*), the system is observable.

(2) Observable matrix $W_{o}$ can be formed as follows:

(10) $$O=\left[\begin{array}{c} C\\ CA\\ \cdots \\ CA^{n-1} \end{array}\right]$$

If ***O*** has full rank *n*, the system is observable.

(3) Another definition of observability involves the observable matrix $W_{o}$; the solution of the Lyapunov equation is as follows:

(11) $$A^{T}W_{o}+W_{o}A+C^{T}C=0$$

defined as

(12) $$W_{o}=\int_{0}^{\infty }e^{A^{T}\tau }C^{T}Ce^{A\tau }d\tau$$

The system is observable if the solution $W_{o}(t)$ is non-singular (determinant is non-zero). The diagonal elements of the observability matrix give information about the relative observability of the different modes.

The observability matrix quantifies the ability to infer the internal state of the system from its outputs [21]. If a perturbation of an optical camera component is highly observable, it means that a change in that variable will significantly affect the output performance of the system.

Based on the state space form of the system, we can derive the closed-loop controllability and observability matrices by substituting the ***A***, ***B***, and ***C*** matrices into the Lyapunov equation. The expressions for the controllability matrix and the observability matrix for the ith order mode are as follows:

(13) $$\mathrm{Controllability}\;\mathrm{matrix}\;\mathrm{diagonal}:\;w_{ci}=\frac{{\left\|B_{i}\right\|}_{2}^{2}}{4\zeta_{i}\omega_{i}}$$

(14) $$\mathrm{Observability}\;\mathrm{matrix}\;\mathrm{diagonal}:\;w_{oi}=\frac{{\left\|C_{i}\right\|}_{2}^{2}}{4\zeta_{i}\omega_{i}}$$

where ${\left\|\;\right\|}_{2}^{2}$ notation represents the Euclidean norm, the square root of the sum of squares of the elements of a vector. $\zeta_{i}$ is the critical damping ratio of the ith order mode, and $\omega_{i}$ is the eigenvalue of the ith order mode. When the input point is *k* and the output point is *j*, the ***B*** and ***C*** matrices of the ith order mode are expressed as follows, in which $F_{k}$ represents the input force at the input point *k*, and $z_{nki}$ and $z_{nji}$ represent the nodal displacements at the input and output points corresponding to the ith order mode.

The ***B*** and ***C*** matrices for mode i with input point k and output point *j* are as follows:

(15) $$B=\left[\frac{0}{F_{k}z_{nki}}\right],C=\left[\begin{array}{cc} z_{nji} & 0 \end{array}\right]$$

Substituting the ***B*** and ***C*** matrix expressions for the ith order modes into the Equations (13) and (14):

(16) $$w_{ci}=\frac{{\left\|B_{i}\right\|}_{2}^{2}}{4\zeta_{i}\omega_{i}}=\frac{{\left\|\left[\frac{0}{F_{k}z_{nki}}\right]\right\|}_{2}^{2}}{4\zeta_{i}\omega_{i}}=\frac{F_{k}^{2}z_{nki}^{2}}{4\zeta_{i}\omega_{i}}$$

(17) $$w_{oi}=\frac{{\left\|C_{i}\right\|}_{2}^{2}}{4\zeta_{i}\omega_{i}}=\frac{{\left\|\left[\begin{array}{cc} z_{nji} & 0 \end{array}\right]\right\|}_{2}^{2}}{4\zeta_{i}\omega_{i}}=\frac{z_{nji}^{2}}{4\zeta_{i}\omega_{i}}$$

Modes can be sorted through Equation (18) according to the matrix of controllability and observability of each mode. The input force of the input point *k* is independent of the mode order, so it does not need to be considered in modal sorting.

(18) $$\frac{z_{nki}^{2}z_{nji}^{2}}{16\zeta_{i}^{2}\omega_{i}^{2}}$$

### 3.2. Balance Reduction

Balanced reduction is a method to reduce the complexity of a dynamic system model. It significantly decreases the dimensionality of the system model by preserving the essential dynamic behavior while minimizing the influence of unimportant modes [22]. This approach is particularly beneficial for the design and analysis of opto-mechanical systems, as lower-dimensional models are more computationally efficient and facilitate effective control.

By understanding the controllability and observability of the system, a new system is constructed that maintains the same diagonal controllability and observability through the application of Hankel singular value decomposition. Given that the diagonal terms of the two matrices are equivalent, both can be utilized to order the states for reduction. Consequently, the ambiguity that may arise from relying solely on energetic control or energetic observability is resolved.

The controllability and observability matrices are positive definite. When a linear transformation is applied to the state vector *x*, the dynamic properties and eigenvalues of the system remain invariant, provided that the transformation matrix is of full rank. However, the controllability and observability matrices undergo changes during linear transformation. A transformation matrix T exists such that the diagonal entries of the controllability and observability matrices are equal; in this scenario, the state space model is deemed balanced.

In accordance with the Lyapunov equation, Cholesky matrix decomposition is performed on the controllability matrix $W_{c}$ and the observability matrix $W_{o}$:

(19) $$W_{c}=L_{c}L_{c}^{T}$$

(20) $$W_{o}=L_{o}L_{o}^{T}$$

The matrices $L_{c}$ and $L_{o}$ are lower triangular matrices that can be obtained directly without actually calculating $W_{c}$ and $W_{o}$. Meanwhile, it is also possible to obtain the $W_{c}$ and $W_{o}$ of the system based on the Lyapunov equations and then use the Cholesky matrix decomposition. In Equation (21), singular value decomposition is performed on the product of the Cholesky triangular matrices to determine ***U***, ***Λ***, and ***V***, where the matrix ***Λ*** is a diagonal matrix:

(21) $$L_{o}^{T}L_{c}=U\Lambda V^{T}$$

Create the transformation matrix ***T***:

(22) $$T=L_{c}V\Lambda^{-1/2}$$

Then, system matrix $A_{b}$, input matrix $B_{b}$, and output matrix $\;C_{b}$ of the balanced system are determined:

(23) $$\begin{array}{l} A_{b}=T^{-1}AT\\ B_{b}=T^{-1}B\\ C_{b}=CT \end{array}$$

Substituting Equation (23) into Equation (6) gives the state space equations for the balanced system:

(24) $$\begin{array}{l} {\dot{x}}_{b}=TAT^{-1}x_{b}+TBu\\ y=CT^{-1}x_{b}+Du \end{array}$$

The controllability matrix $W_{c}$ and the observability matrix $W_{o}$ are also converted from the matrix ***T*** to the same diagonal form:

(25) $$W_{bo}=W_{bc}=diag(g)$$

Since the controllability and observability matrices are the same, there is no ambiguity in deciding whether controllable or observable modes should be selected. The state terms to be retained are the largest number of diagonal terms.

Due to the mass-limited stiffness of the optical camera, the opto-mechanical structures are usually damped relatively low; such structures are prone to significant resonance and trigger complex dynamic behavior. Through controllability and observability, the balanced reduction method can accurately capture the main dynamic modes associated with resonance and significantly simplify the model without substantially degrading its accuracy. This not only improves solution efficiency but also provides a deeper understanding of the dynamic properties of the system.

## 4. Simulation Analysis and Test

### 4.1. Modal Analysis

**Figure 3:**
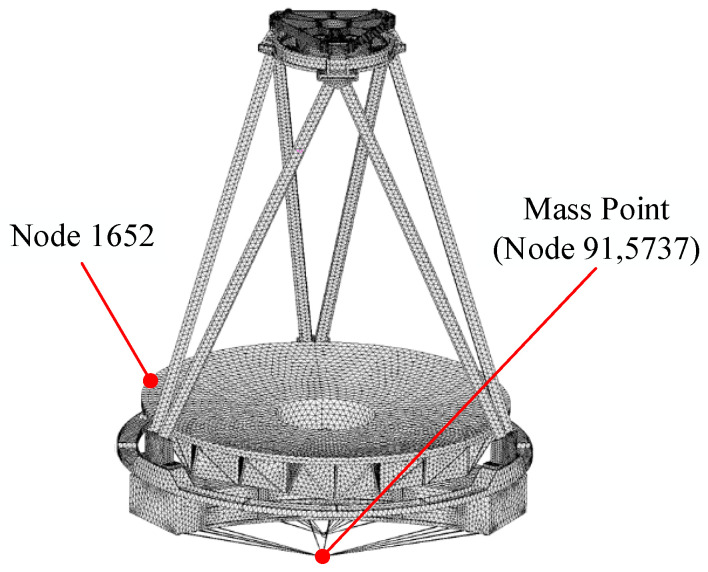
Finite element modelling of optical remote sensing cameras.

Modal analysis is the basis of structural dynamics analysis. Through modal analysis, the natural frequency and modal mode of the optical camera can be solved, and the structural vibration form of the whole camera can be obtained. In this paper, the finite element software HyperMesh version 2017 (Altair, Troy, MI, USA) and MSC Patran version 2010 (MSC Software, Los Angeles, CA, USA) are used to mesh the whole camera model, set the boundary conditions, and assign the material properties of the mesh. The FEM of the whole machine is shown in Figure 3, which contains 459,064 elements and 866,825 nodes. During the simulation analysis, in order to simulate the disturbance exerted by the shaker on the optical camera, node 915,737 was used to replace the shaker as the excitation input point. Under the action of external excitation, the middle position between any two support points on the mirror can be simplified into a cantilever beam model. Therefore, node 1652, which is located at the middle position between any two support points of the primary mirror, was selected as the response output point. In the finite element modeling process, we are more concerned with the structural dynamics characteristics of the primary mirror assembly, secondary mirror assembly, and truss assembly. Therefore, the corrector mirror assembly, the focusing mechanism, and the focal plane electrical box are simplified as mass points, and these mass points are connected to their mounting surfaces by RBE2.

**Figure 4:**
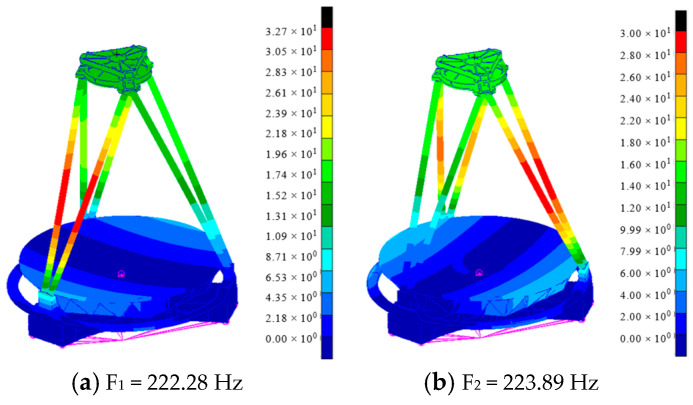

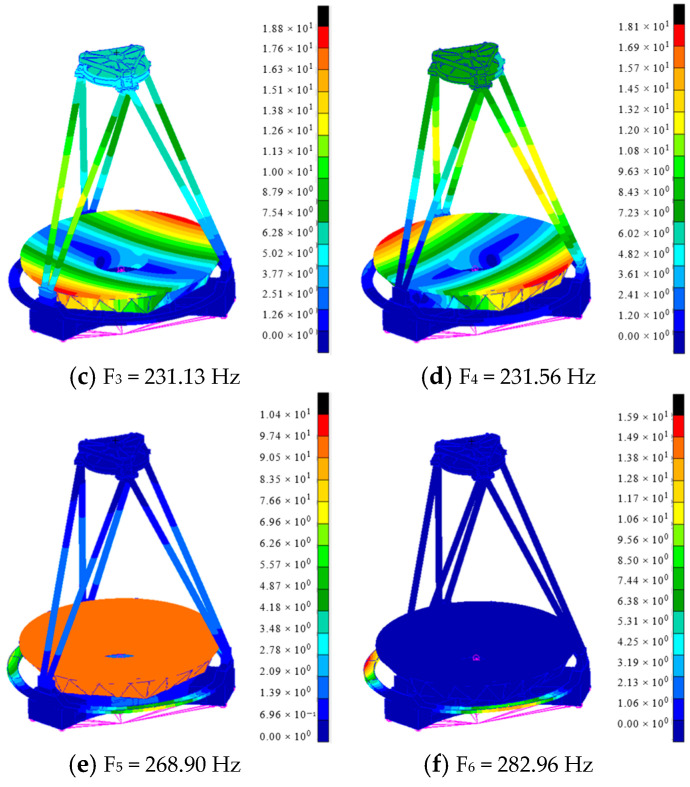
Modal shapes of the first 6 modes.

Modal analysis of the whole camera FEM was performed using MSC/NASTRAN (MSC Software, Los Angeles, CA, USA). In order to simulate the excitation of the shaker on the optical camera during the vibration test, the analysis is carried out using the large mass point to simulate the vibration of a single DOF. To ensure the accuracy of the calculation results, the large mass point should be set to 10^3^–10^8^ times the mass of the structure [15]. When the large mass point is set to 10^6^ times the mass of the whole camera, it can stimulate not only the structural response of the whole camera but also the excitation of the shaker on the whole camera. Therefore, a large mass point of 10^6^ times the mass of the whole machine is established. The mass point and the whole camera are constrained in a multi-point control manner. Considering the rigid connection between the whole camera and the shaker, RBE2 and MPC were used to connect the large mass point to the nodes of the primary mirror substrate mechanical mounting interface. During the test, the connection between the shaker and the camera remains constant, and there is no relative motion between them. Therefore, the boundary conditions are constrained. The large mass point is constrained by six DOF, and the first 50 natural frequencies of the whole camera are calculated. Table 1 demonstrates the first six orders of intrinsic frequencies of the whole machine. Figure 4 shows the first six modal shapes of the whole machine.

As shown in Figure 4, the first order mode of the whole camera is 222.28 Hz, which characterizes the vibration of the truss rod around the Y-axis direction. The second order mode of the whole camera is 223.89 Hz, which characterizes the vibration of the truss rod around the X-axis direction. The third order mode of the whole camera is 231.13 Hz, which characterizes the oscillation of the primary mirror assembly around the Y-axis direction. The fourth order mode of the whole camera is 231.56 Hz, which characterizes the oscillation of the primary mirror assembly around the X-axis direction. The fifth order mode of the whole camera is 268.90 Hz, which characterizes the translation of the primary mirror assembly along the Z-axis direction. The sixth order mode of the whole camera is 282.96 Hz, which characterizes the oscillation of the truss around the X-axis direction. According to the modal analysis, the whole camera has a large stiffness, and the first order modal is 222.28 Hz, which can avoid structural resonance in the normal working process.

### 4.2. Frequency Response Analysis with Finite Element Method

The frequency transfer characteristic of the optical camera is the maximum steady state response of the whole camera to a simple harmonic excitation in the frequency domain as a function of the frequency of the excitation. The frequency transfer characteristic can reflect the response of the whole camera to the external excitation, which is a kind of structural dynamics analysis. Usually, the frequency response characteristics of the whole camera are carried out by the finite element method.

**Figure 5:**
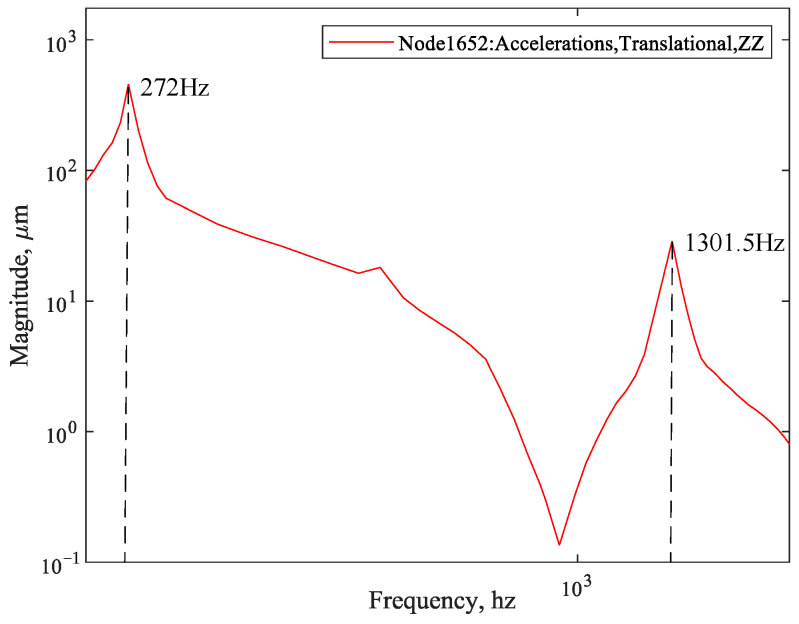
Frequency response curve of the Finite Element Analysis.

The frequency response analysis is carried out by using finite element software. According to the results of the modal analysis, the frequency range is set to (200 Hz, 2000 Hz), and the frequency step is 36 Hz. Based on the previous engineering experience and test results of the research group, the structural damping was revised through the dynamic amplitude–frequency response of the acceleration of the dynamic experiments. The dynamic amplification coefficient of the system is estimated to be 50.3. *Q* = 1/*ξ* (where *ξ* is the structural damping coefficient). The structural damping coefficient value *g* = 0.02 is calculated. The frequency response analysis of the FEM of the whole camera is carried out using the modal method in MSC/NASTRAN (MSC Software, Los Angeles, CA, USA). The modal mode shape of the structure is used to reduce and decouple the coupled equations of motion. Through the established large mass points (nodes 915,737), the whole camera is excited with a 0.2 g acceleration frequency response, and the boundary conditions are constrained. The middle position of any two support points of the machine is chosen as the response output point, node 1652. The analysis results are shown in Figure 5.

### 4.3. Frequency Response Analysis with State Space Method

Faced with large and complex structural models, the finite element method involves a huge amount of computation and takes a long time to solve. In addition, the limited functionality of finite element software does not facilitate the observation of the contribution of each mode to the overall response and does not always meet the needs of the structural design optimization of opto-mechanical structures. To improve the solution efficiency, the state space method can be used to calculate the structural frequency response problem. According to the results of modal analysis, the frequency range of frequency response analysis is determined to be [220 Hz, 2000 Hz], the damping form is modal damping *g* = 0.02, and the state space model of the whole camera is established by taking the frequency transmission characteristics between node 915,737 and node 1652 as an example. Among them, node 915,737 is the excitation input point, while node 1652 is the response output point.

The analysis steps to carry out dynamics modelling based on the state space method as well as model reduction are as follows:

(1) Data acquisition. Perform modal analysis on the finite element model of the whole camera; extract the natural frequency of the appropriate order modal data from the obtained result file; extract the eigenvalues of the system, as shown in Table 2; extract the node displacements of the excitation input point and the response output point, and generate the modal matrix (take the Z-direction displacement as an example, Equation (26)); and determine the frequency range based on the results of the modal analysis.
(2) State space model establishment. Read the data file, establish the state space model of the camera, and plot the frequency transfer characteristic curve.
(3) Modal reduction. Use Equations (9) and (12) to solve the controllability and observability matrices for each non-rigid body mode, and then, use the transformation matrix T to make the diagonal terms of the controllability and observability matrices equal. Sort the matrix diagonal entries from largest to smallest. Modes with non-zero diagonal entries are retained, modes with zero diagonal entries are reduced, and the state space model is regenerated from the retained modes to obtain the modal reduced system model.
(4) Modal reduction results. The frequency response of the simplified system model is calculated to obtain the frequency transfer characteristic curves of nodes 915,737–1652.

$x_{n}$ is the system modal matrix as shown in Equation (26), which consists of the Z-direction displacements of the excitation input points and the response output points, and these displacement data can be obtained in the f06 file after the modal analysis.

(26) $$\begin{array}{ll} x_{n}= & \left[\begin{array}{cc} Z_{1\_node\_input} & Z_{n\_node\_input}\\ Z_{n\_node\_output} & Z_{n\_node\_output} \end{array}\right]\begin{array}{c} \leftarrow \\ \leftarrow \end{array}\begin{array}{c} DOF_{1}\\ DOF_{n} \end{array}\\ & \begin{array}{ccccccc} & & ↑ & & & & ↑\\ & & Mode1 & & & & Mode2 \end{array} \end{array}$$

The frequency response curves of nodes 915,737–1652 are solved based on the state space model of the whole camera. The state space method and finite element method are used to analyze the frequency transfer characteristics of the whole camera. In the state space method, the DC gain method and the balance reduction method are used to reduce the mode order of the state space model for comparison. The DC gain method is used to obtain the contribution of each order mode to the overall frequency response, the DC gain value, and rank them from large to small, retaining the modes corresponding to larger DC gain values.

**Figure 6:**
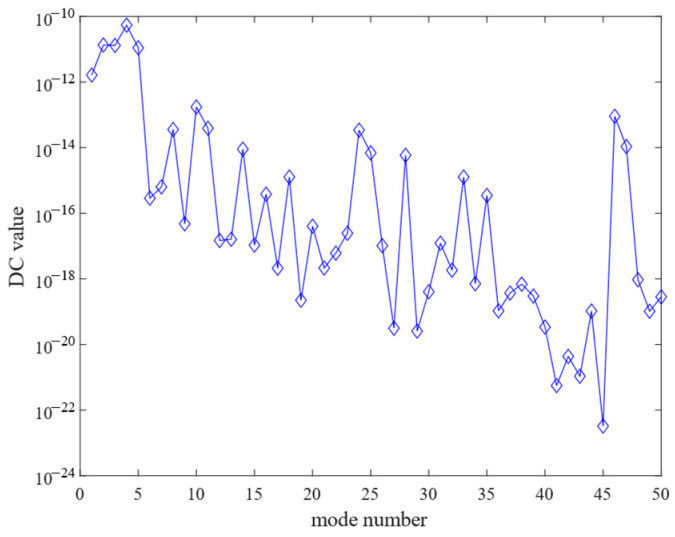
DC value of each mode contribution versus mode number.

**Figure 7:**
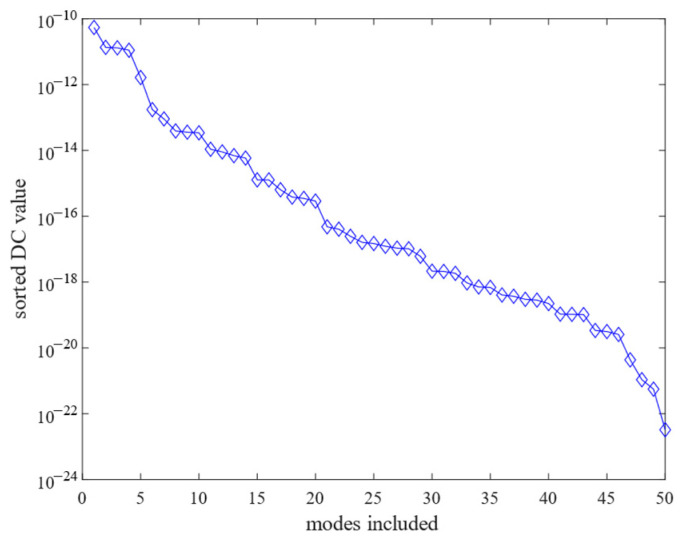
Sorted DC value of each mode versus number of modes included.

**Figure 8:**
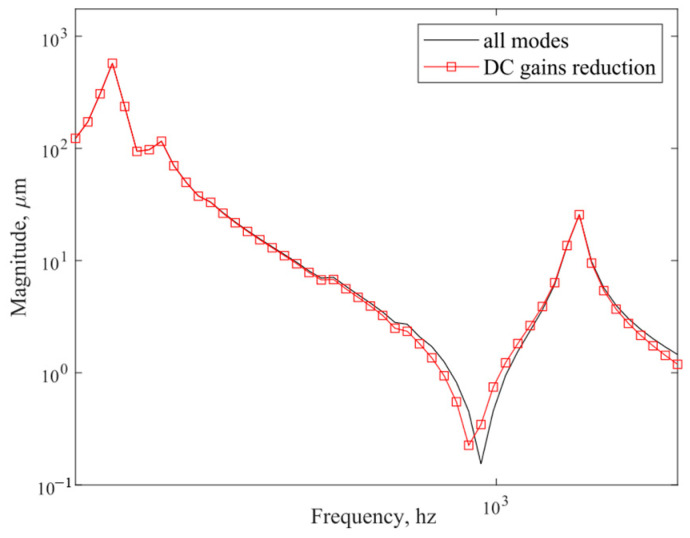
Comparison of the frequency response curves of all modes and DC gain reduced to 15th order modes.

Figure 6 illustrates the DC gain contribution values for each order of modes. Figure 7 sorts the DC gain contribution values for each order of modes from largest to smallest. Different numbers of mode orders were retained, respectively, and after comparison, it was found that the frequency response curve when 15 modes were retained was basically consistent with that when all modes were retained. Figure 8 compares the frequency response curves for all modes with the DC gain after reduction to 15th order modes. The comparison reveals that the frequency response curves of the reduced system do not exactly coincide with the unreduced ones.

**Figure 9:**
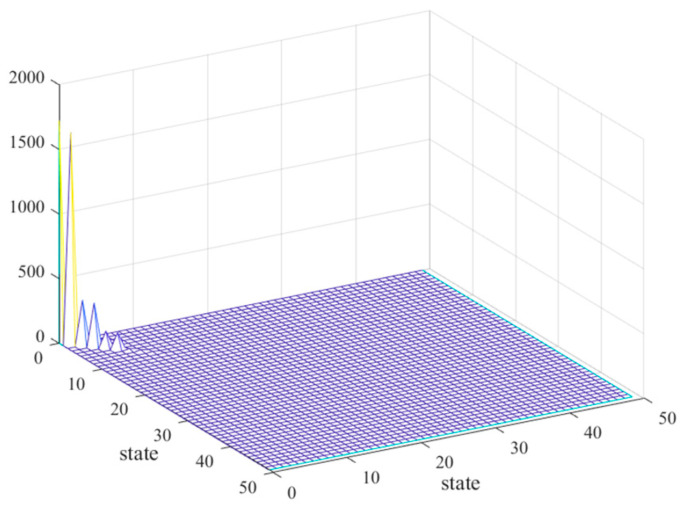
Controllability/observability matrix of balanced system.

**Figure 10:**
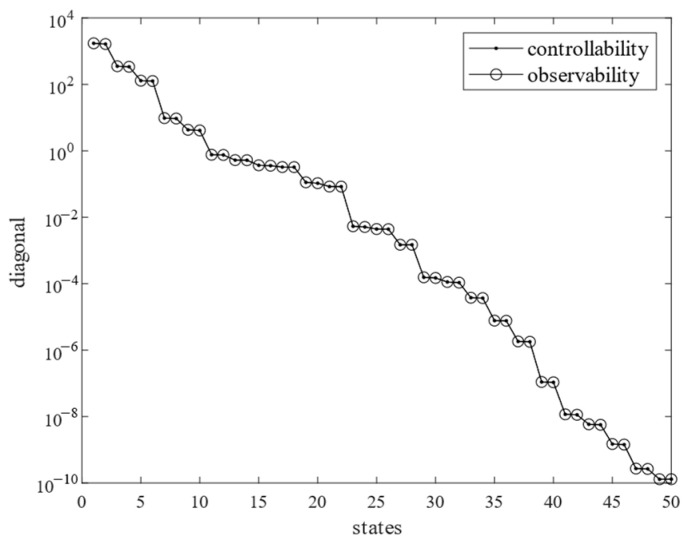
Controllability and observability matrix diagonal terms of balanced system.

**Figure 11:**
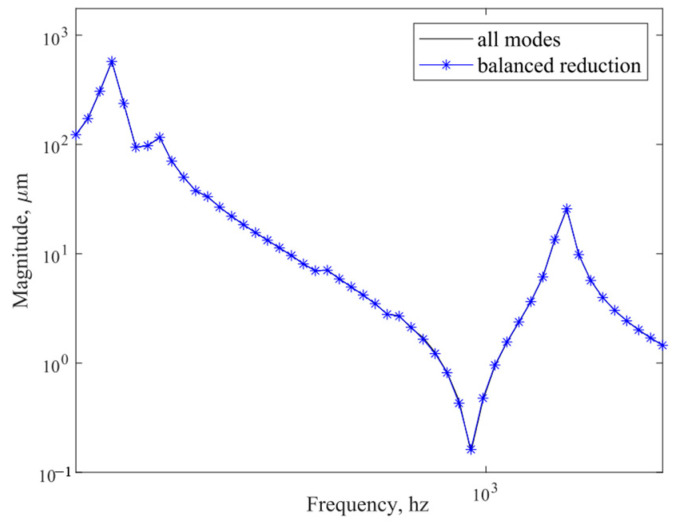
Comparison of the frequency response curves of all modes and balanced reduced to 15th order modes.

Figure 9 plots the controllability/observability matrices for the balanced system, where controllability and observability matrices are identical and strictly diagonal. Figure 10 plots the controllability and observability matrix diagonal terms of the balanced system for modal reduction. According to Figure 9 and Figure 10, we see that, except for the first 15 orders of the diagonal terms, the rest of the orders of the diagonal terms are close to 0, which do not contribute to the response of the whole camera, so only the first 15 orders of the modal terms are retained. Figure 11 compares the frequency response curves for all modes with the balanced reduced to 15th order modes. The comparison shows that the frequency response curves of the reduced system are basically the same as the unreduced one.

**Figure 12:**
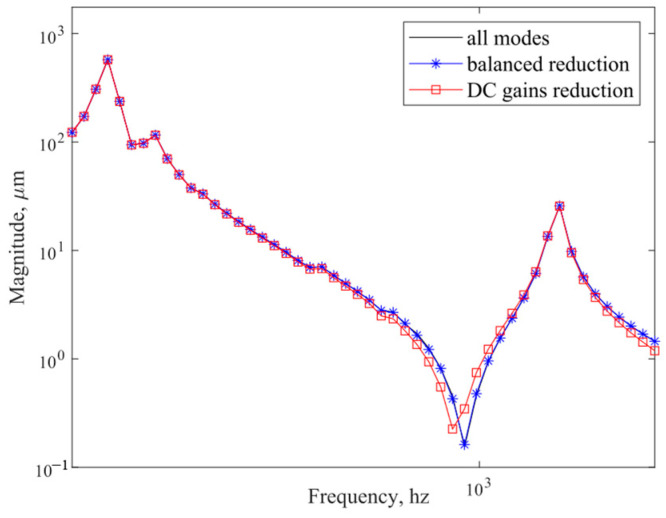
Frequency response curves after all modes are compared with DC gain reduction and balanced reduction.

In Figure 12, the frequency response curves obtained from the DC gain modal reduction of the state space method and the simulation after the balanced reduction are compared. The state space method performs the balanced reduction, and only the 15th order modes that contribute to the system are retained for the analysis; therefore, the curves do not overlap completely.

**Figure 13:**
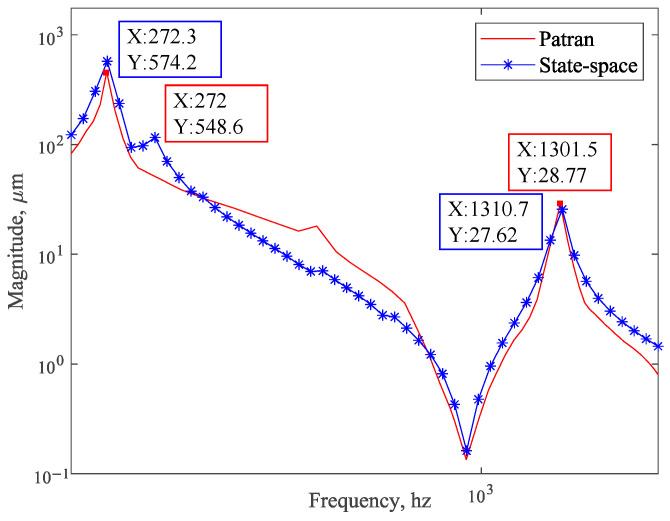
Frequency response curve comparison between finite element analysis (Patran) and state space methods.

According to Figure 12, the frequency response curve obtained by the balanced reduction method has the highest similarity with the unreduced frequency response curve. Therefore, in Figure 13, the frequency response curve obtained by the balanced reduction method is compared with the curve obtained using finite element analysis (Patran). The comparison results show that the two curves are not exactly coincident due to the reduction of the system model by the state space method.

The results show that the frequency response curves have the same linear trend over the frequency range, and the peaks and troughs of the curves correspond to the same frequency points. There are errors in the low-frequency response curves due to the different fitting algorithms for the frequency response characteristic curves by Patran and the state space method proposed in this paper. The camera is prone to damage and plastic deformation at the resonance peak, so we are only concerned with the response of the structure at the resonance peak. The relative errors of the frequency response curves obtained by the two methods at the peak resonance response are 4.67% and 3.99%, which are within the permissible range according to engineering experience. Therefore, the analysis by the state space method is accurate.

There are two different analysis methods for frequency response analysis: the direct method and the modal method. The direct method solves the coupled equations of motion directly at a given frequency, while the modal method uses the modal shapes of the structure to reduce and decouple the coupled equations of motion and then obtains the response at a given frequency by superposition of individual modal responses. Since the modal frequency response method can only be used to decouple the equations when there is no damping or only modal damping, the direct method is used in this paper. As shown in Table 3, the state space method simplifies the model by reducing the modal frequency response, which can improve the computational efficiency.

Accurate frequency transfer characteristics of the optical camera can be obtained by using the finite element method analysis. However, the whole analysis process takes approximately 20 min. While the balanced reduction method of state space characterization only needs about 20 s to provide the frequency response curve of the system, its computational efficiency is better than the finite element method. It can obtain the controllability and observability of the modes in each order, which is more convenient for the design and analysis of the structure of the optical camera.

### 4.4. Dynamic Vibration Test of the Whole Machine

**Figure 14:**
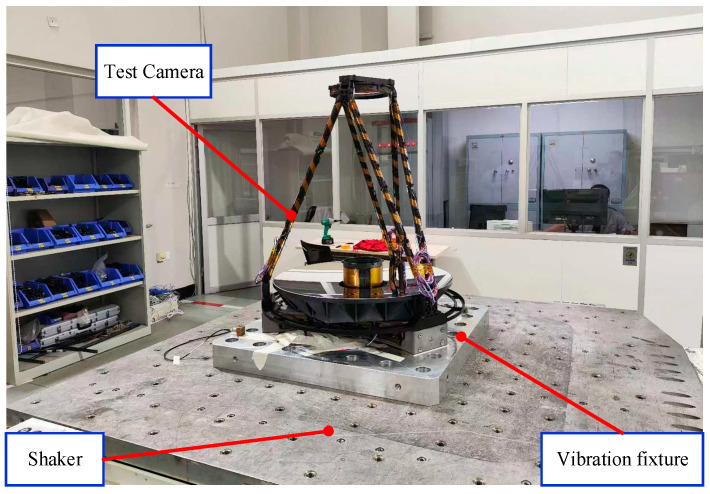
Vibration test of optical camera.

In order to understand the real dynamics of the whole camera and verify the results with the theoretical simulation analyses, the swept frequency vibration test was carried out for the optical camera. As shown in Figure 14, the camera is attached to the shaker through the vibration fixture, and the vibration tests are carried out in an environmental test station. The whole camera and shaker are connected by steel screws through a vibration fixture, and the connection relationship remains unchanged during the test. The shaker performs a sinusoidal sweep of 0.2 g in the X, Y, and Z directions on the camera. The sinusoidal sweep results can not only reflect the modal frequency of the camera and verify the accuracy of the simulation analysis results but also determine whether there exists a phenomenon that the response amplification is too large, which may cause structural damage to the camera during the test through the peak of the curve.

**Figure 15:**
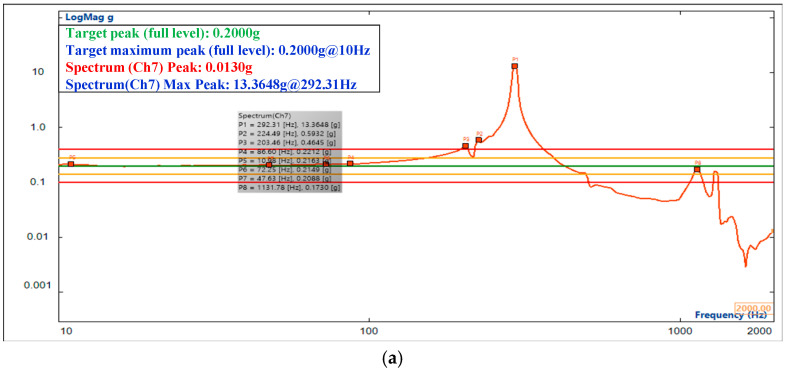

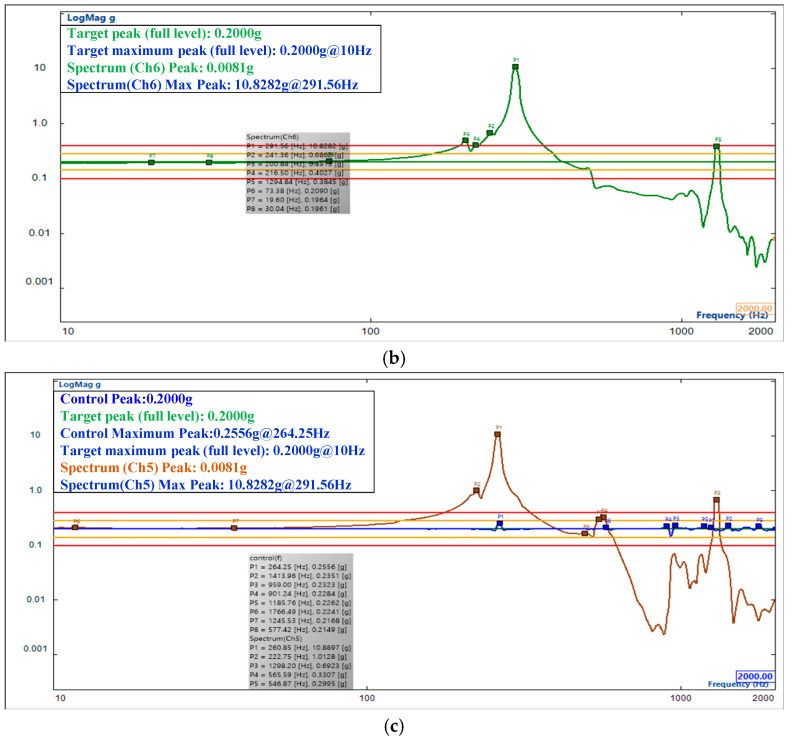
Results of sine sweep test. (**a**) X-direction sine sweep test; F_x_ = 292.31 Hz. (**b**) Y-direction sine sweep test, F_y_ = 291.56 Hz. (**c**) Z-direction sine sweep test; F_z_ = 260.85 Hz.

Sweep tests were performed in the range of 10–2000 Hz. There is an error between the theoretical model and the actual structure due to the process of finite element modeling, which simplifies part of the structure to mass points. However, as shown in Figure 15, the frequency response curves and peak responses are the same. In the X direction, the tested value of the first-order natural frequency of the camera is 292.31 Hz, and the natural frequency analyzed by simulation is 308.0 Hz, with a relative error of 5.37%. In the Y-direction, the tested value of the first-order natural frequency of the camera is 291.56 Hz, and the natural frequency analyzed by simulation is 308.0 Hz, with a relative error of 5.64%. In the Z-direction, the test value of the first-order natural frequency of the camera is 260.85 Hz, and the natural frequency analyzed by simulation is 272.0 Hz, with a relative error of 4.27%. The frequency response curve of the simulation analysis has approximately the same trend as the sweep test curve. The relative error of the natural frequency is small in the X, Y, and Z directions, which proves that the simulation analysis results of the whole camera are accurate. The connection reliability of the camera is high, and the dynamic stiffness is good in all three directions.

## 5. Discussion

Based on the state space theory and structural dynamics theory, the dynamic characteristics of the space optical camera with an aperture of φ572 mm are analyzed, a linear state space model of the whole camera is established, and the modal reduction is carried out by using the balanced reduction method. The frequency response analysis is carried out on the reduced state space model, and the frequency response curves between the excitation input point and the response output point are obtained. The initial frequency response curves are plotted and compared with the response curves of the DC gain method and the balanced reduction method. The comparison reveals that the results of the reduced model and the initial model are closer, which proves that the method can simplify the calculation and analysis process while guaranteeing the accuracy.

Finally, the structural reliability of the whole camera and the accuracy of the simulation analysis are verified by the mechanical vibration test of the whole camera. The simulation results and test results are shown in Table 4, indicating that the accuracy of the simulation analysis in this paper is high. This paper provides a new method for dynamic analysis of the opto-mechanical structures. The method is simple and flexible, which greatly improves computational efficiency and ensures better accuracy. It can be widely used in the design and analysis of opto-mechanical structures.

## 6. Conclusions

This article presents an efficient and accurate dynamic analysis method for lightweight opto-mechanical structures. Its core is to establish the state space model of the whole camera by using structural dynamics and linear state space theory and to perform modal reduction and frequency response analysis based on the state space model. Through the controllability and observability concepts in control theory, the order of the model is reduced, and the frequency response of the reduced state space model is analyzed. The response curves obtained by the traditional finite element method, the DC gain method, and the balance reduction method are compared, and their peak resonant responses are within 5% error. It is proved that the method can simplify the calculation and analysis process while ensuring accuracy. The accuracy and rationality of the simulation analysis are verified by dynamic experiments.

## Figures and Tables

**Table 1:** Natural frequency of the first 6 orders of the whole camera.

Order	F_1_	F_2_	F_3_	F_4_	F_5_	F_6_
Frequency	222.28	223.89	231.13	231.56	268.90	282.96

**Table 2:** Modal information in the result file.

Mode	Frequency	Eigenvalue
1	2.222793 × 10^2^	1.950553 × 10^6^
2	2.238885 × 10^2^	1.978898 × 10^6^
3	2.311281 × 10^2^	2.108945 × 10^6^
4	2.315579 × 10^2^	2.116795 × 10^6^
5	2.688967 × 10^2^	2.854503 × 10^6^
6	2.829597 × 10^2^	3.160886 × 10^6^
7	2.832125 × 10^2^	3.166536 × 10^6^
8	2.841080 × 10^2^	3.186594 × 10^6^
9	2.873277 × 10^2^	3.259227 × 10^6^
10	3.274861 × 10^2^	4.233947 × 10^6^

**Table 3:** Comparison of computational efficiency between finite element method and state space method.

Tool	MSC Patran/Nastran	State Space Model
Analytical process	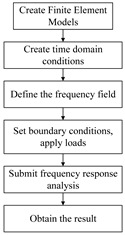	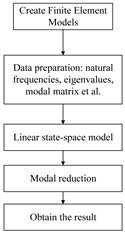
Consuming time	20 min	20.036 s

**Table 4:** Comparison between experimental test results and simulation results.

	X Direction		Y Direction		Z Direction	
	Fundamental Frequency	Relative Error	Fundamental Frequency	Relative Error	Fundamental Frequency	Relative Error
FEA simulation	308 Hz	5.37%	308 Hz	5.64%	272 Hz	4.27%
Test	292.31 Hz		291.56 Hz		260.85 Hz	

## Data Availability

The data that support the findings of this study are included and are available from the corresponding author.
